# Successful Treatment of Brevibacterium Bacteremia Solely With Antimicrobial Therapy

**DOI:** 10.7759/cureus.16004

**Published:** 2021-06-28

**Authors:** Charles E Benson, Luis Tatem

**Affiliations:** 1 Infectious Diseases, State University of New York Downstate Medical Center, New York, USA

**Keywords:** brevibacterium, bacteremia, central venous catheter infection, immunocompromised, picc line

## Abstract

*Brevibacterium* is a large genus that is not often involved in pathogenesis, however, since 1991 there have been several case reports of *Brevibacterium*-associated illness, most often due to bacteremia in the setting of an immunocompromised patient with a central venous catheter (CVC). Here we detail the case of an elderly woman with many comorbidities and a peripherally inserted central catheter (PICC) line for over four years, who presented with septic shock and *Brevibacterium* bacteremia. In nearly all previous cases of *Brevibacterium* bacteremia it was thought to be due to a CVC which was removed as part of the treatment in conjunction with antibiotics. In this case, the patient was treated with empiric antibiotics and her blood cultures cleared within 48 hours without catheter removal or antibiotic-lock therapy. The clinical outcome was favorable at 50 days follow-up.

## Introduction

The genus of *Brevibacterium* contains 50 different species, nine of which have been isolated from humans, including *B. casei* (most common), *B. epidermidis, B. otitidis, B. paucivorans, B. sanguinis, B. linens, B. iodinum, B. mcbrellneri, and B. massiliense* [1]. Though this genus has been found on human skin, it was thought to be apathogenic until the discovery of a patient with *Brevibacterium* bacteremia in 1991. Since then, various case reports of bacteremia most often associated with central venous catheters in patients with malignancy or with immunocompromised conditions have been published. Although uncommon, they are important and under-recognized etiologic agents of infections in immunocompromised patients [2].

## Case presentation

**Figure 1:**
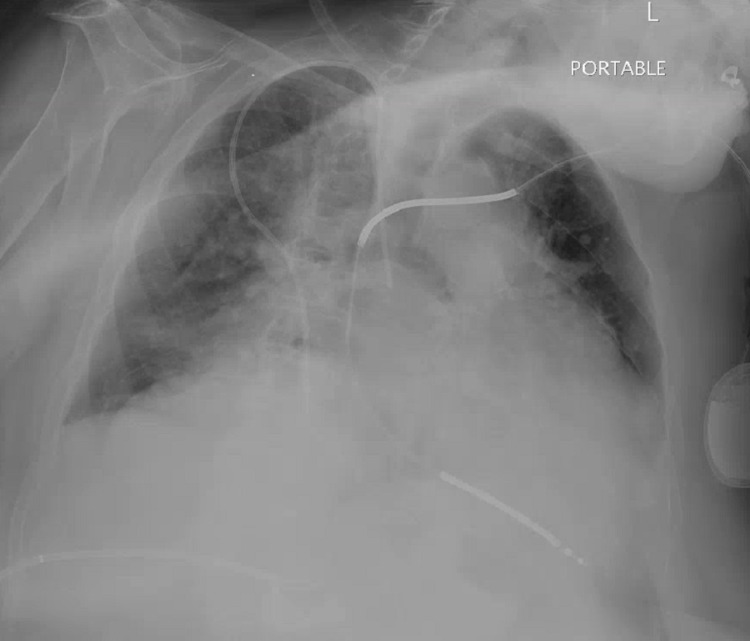
Portable chest X-ray antero-posterior (AP)

**Figure 2:**
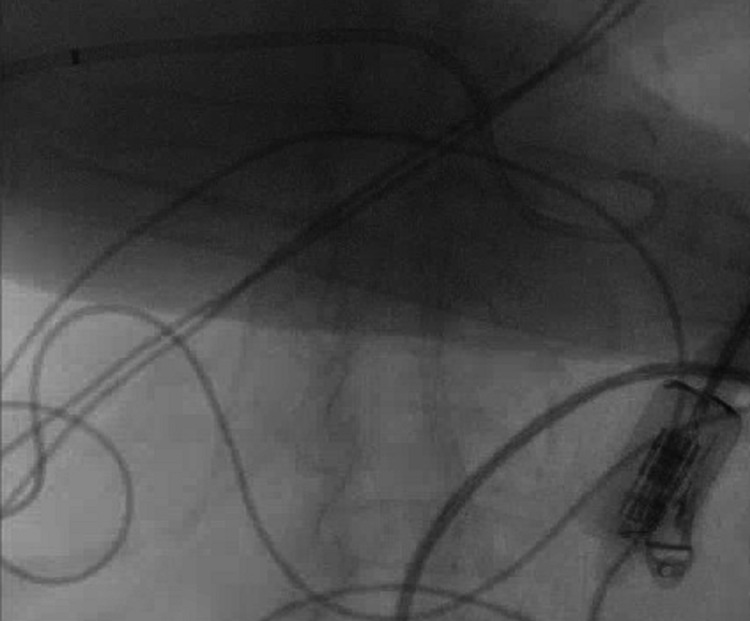
Cholangiogram showing the percutaneous cholecystostomy tube (PCT) withdrawn

An 85-year-old female with a past medical history of cholangitis and common bile duct (CBD) stones, who has had a percutaneous cholecystostomy tube (PCT) since 2018 due to her high surgical risk, heart failure reduced ejection fraction (HFrEF) on home intravenous milrinone via right subclavian peripherally inserted central catheter (PICC) (Figure 1) which was placed four years prior and never exchanged, implantable cardioverter defibrillator (ICD), coronary artery disease, aortoiliac aneurysm and mural thrombi, presented with altered mental status and fever of 100.9 F. Initial vitals showed a heart rate of 132 bpm, systolic BP of 100 mm Hg, and an SpO2 of 91% on room air which improved to 95% with 15 L of non-rebreather. On exam she was noted to be disoriented, mildly dyspneic and had decreased air entry bilaterally, her right-sided PICC line site was clean without erythema, induration, or discharge, same as with her ICD site. Heart sounds were positive without any murmurs, abdominal exam showed leakage around her PCT and decreased drainage into the biliary collecting bag. She initially received two liters of intravenous fluids, was started on a norepinephrine drip due to worsening hemodynamic instability and received 2 g of ceftriaxone plus 500 milligrams of metronidazole. A set of blood cultures were obtained, a urinalysis was negative for leukocyte esterase or nitrates, and her white blood cell count was 7.45 K/mL with 25% bands on manual differential. A cholangiogram was done showing the catheter to have withdrawn into the common bile duct (Figure 2). The CBD was moderately dilated with several filling defects consistent with calculi and a biliary drainage catheter exchange was done. Computer tomography of the abdomen showed enhancement of gallbladder wall, but the family declined endoscopic retrograde cholangiopancreatography (ERCP) and declined PICC line exchange. She was given vancomycin, cefepime, and metronidazole for empirical coverage of her sepsis syndrome as she was clinically unstable and was continued on norepinephrine support. Bile cultures from a previous drainage system showed *Aeromonas, Escherichia coli, Klebsiella pneumoniae, Enterococcus faecalis, and Enterococcus faecium* deemed to be colonizers as they were obtained from the drainage system, the polymicrobial nature of the specimen and as she had clinically stabilized despite the lack *E. faecium* coverage. Initial blood cultures revealed *Brevibacterium* species in the aerobic bottle after 96 hours of incubation, identified with matrix-assisted laser desorption ionization-time of flight mass spectrometry (MALDI-TOF MS) and a follow-up blood culture in 24 hours again showed a similar isolate. Antibiotics were switched to ceftriaxone 2g daily plus metronidazole to cover for her gallbladder infection and she was continued on intravenous vancomycin empirically for *Brevibacterium* bacteremia. After 48 hours her condition completely stabilized, and cultures cleared on the third day of admission. She received a total of 10 days of intravenous vancomycin and this was followed by oral doxycycline as* *she was allergic to penicillins; quinolones were not ideal due to her advanced age and aortic aneurysm. The family did not want another IV access and her PICC line was exclusively used for her milrinone continuous infusion, therefore this non-standard approach was performed. *Brevibacterium* antimicrobial susceptibility became available after discharge: Benzyl penicillin MIC 0.50 ug/ml intermediate, ciprofloxacin MIC 0.47 ug/ml susceptible, erythromycin MIC 0.50 ug/ml susceptible, gentamicin MIC 0.064 ug/ml susceptible, tetracycline MIC 16 ug/ml resistant, trimethoprim/sulfamethoxazole >32 ug/ml resistant, and vancomycin 0.50 ug/ml susceptible, according to the Clinical and Laboratory Standards Institute guidelines for corynebacteria [3]. We decided to stop her antibiotics seven days after discharge as the isolate was resistant to tetracyclines and follow her closely. She remains stable 60 days after discharge without relapse of her *Brevibacterium* bacteremia and without the need for a catheter exchange, without requiring further antibiotic therapy; after two months catheter was exchanged over the wire as per family request.

## Discussion

*Brevibacterium *are gram-positive rods, obligate aerobic organisms that are irregular and resemble corynebacteria. They are most often associated with milk products where they contribute to aroma and color but they are also found on human skin, genital hair, and otorrhea [2]. *Brevibacterium* is rarely a pathogenic genus, the first case not being discovered until 1991 when *B. epidermidis* was found to be the cause of a central line-associated bloodstream infection [4]. Since this discovery, it has infrequently been found as a pathogen with a total of 18 cases reported implicating *Brevibacterium*, the most common infection being bacteremia (10 of these cases). The most common species that is isolated is *B. casei* [5]. There have also been cases of peritonitis, pericarditis, endocarditis, brain abscess, and osteomyelitis [5-17]. As Asai et al. reported, the cases of *Brevibacterium* bacteremia most often occur as opportunistic pathogens in patients who are immunocompromised, usually due to malignancy or AIDS. Other important risk factors include indwelling foreign materials, prosthetic heart valves, and continuous ambulatory peritoneal catheters for dialysis [18]. There is also an overwhelming correlation between bacteremia by this genus and indwelling central venous catheters (CVC) with a CVC being present in 10 out of 11 cases of bacteremia [2]. Of the cases in which bacteremia is associated with indwelling catheters, all of them were treated with antibiotics for two to three weeks, predominantly with glycopeptides and quinolones - as well as with removal of the patient’s catheter, with some studies recommending this as a preferred part of the treatment, especially in patients with complicated bacteremia [17,19].

It has also been shown in at least one patient that antibiotic-lock therapy with vancomycin, combined with intravenous antibiotic therapy was enough to clear* Brevibacterium* in a patient with uncomplicated bacteremia due to a Hickman catheter [19]. Most of the patients for which the information is available improved with antibiotic therapy and catheter removal, however it is important to note that in one study, 30% of patients had a recurrence of bacteremia from 13 to 28 days [2]. Our patient, while not immunocompromised, had multiple comorbidities and a long-standing CVC. By administering intravenous vancomycin, she was able to clear her bacteremia in two days without the need for catheter removal or antibiotic-lock therapy. Even though her cultures were not obtained directly from the catheter, the fact that the PCT cultures did not isolate the same organism and the presence of a long standing PICC line (over four years) makes this source the most plausible etiology of the *Brevibacterium* bacteremia. The fact that she remains stable 60 days post-discharge makes a catheter retention strategy as an alternative in patients needing to keep their access on a long-term basis and who have poor tolerance for repeated surgical procedures or patient's refusal.

## Conclusions

*Brevibacterium* bacteremia, although uncommon, has now been increasingly reported, especially in patients with malignancy or with immunocompromised conditions who have central venous access. In most of the previous cases in literature, removal of the patient’s central venous access is a paramount step in their management. Here we report a case in which *Brevibacterium* bacteremia was successfully treated solely with intravenous vancomycin for 10 days, with the patient remaining stable at 60 days follow-up without relapse.
